# Are racial/ethnic minorities recently diagnosed with diabetes less likely than white individuals to receive guideline-directed diabetes preventive care?

**DOI:** 10.1186/s12913-021-07146-0

**Published:** 2021-10-25

**Authors:** Felippe O. Marcondes, David Cheng, Margarita Alegria, Jennifer S. Haas

**Affiliations:** 1 Division of General Internal Medicine, Massachusetts General Hospital, MA Boston, USA; 2 Biostatistics Center, Massachusetts General Hospital, MA Boston, USA; 3 Disparities Research Unit, Massachusetts General Hospital, MA Boston, USA; 4 Division of General Internal Medicine, MGH, 100 Cambridge St, Suite 1600, MA 02114 Boston, USA

**Keywords:** Diabetes, Prevention, Disparities, race/ethnicity

## Abstract

**Background:**

Diabetes mellitus has reached epidemic proportions in the United States. As the prevalence of diabetes continues to rise, the burden of disease is divided unevenly among different populations. Racial/ethnic disparities in diabetes care are pervasive, including the provision of care for prevention of complications. Prevention efforts should be focused on the time that immediately follows a diagnosis of diabetes. The aim of this study was to assess racial/ethnic differences in the receipt of guideline-directed diabetes care for complication prevention by individuals recently diagnosed with diabetes.

**Methods:**

We used repeated cross-sections of individuals recently diagnosed with diabetes (within the past 5 years) from the National Health Interview Survey from 2011 to 2017. Multivariate regression was used to estimate the associations between race/ethnicity (non-Hispanic White, non-Hispanic Black and Hispanic) and guideline-directed process measures for prevention of diabetes complications (visits to an eye and foot specialist, and blood pressure and cholesterol checks by a health professional - each in the prior year). We assessed effect modification of these associations by socioeconomic status (SES).

**Results:**

In a sample of 7,341 participants, Hispanics had lower rates of having any insurance coverage (75.9 %) than Non-Hispanic Whites (93.2 %) and Blacks (88.1 %; *p*<0.001). After adjustment for demographics, total comorbidities, SES, and health insurance status, Hispanics were less likely to have an eye exam in the prior year (OR 0.80; (95 % CI 0.65-0.99); *p*=0.04) and a blood pressure check (OR 0.42; (95 % CI 0.28-0.65); *p*<0.001) compared to Non-Hispanic Whites. There was no significant effect modification of race/ethnicity by SES.

**Conclusions:**

Hispanics recently diagnosed with diabetes were less likely to receive some indicators of guideline-directed care for the prevention of complications. Lack of insurance and SES may partially explain those differences. Future work should consider policy change and providers’ behaviors linked to racial/ethnic disparities in diabetes care.

## Background

Diabetes mellitus (hereafter referred to as “diabetes”) has reached epidemic proportions in the United States. As of 2018, 26.9 million people (8.2 % of the US entire population) were diagnosed with diabetes.[1] As the prevalence of diabetes continues to rise,[2] the burden of disease is divided unevenly among different populations.

Racial/ethnic disparities in diabetes are a pervasive public health problem. Socioeconomic, biologic, clinical and health system factors rank among the causes for these differences.[3] The United States of America (U.S.) Centers for Disease Control and Prevention (CDC) estimates that in 2017-2018, the age-adjusted prevalence and incidence of diabetes in the United States was 12.5 % and 9.7 per 1,000 persons among Hispanics and 7.5 % and 5.0 per 1,000 persons among non-Hispanic Whites (hereafter called “Whites”).[1] Non-Hispanic Blacks (hereafter called “Blacks”) also had a higher prevalence (11.7 %) and incidence (8.2 per 1,000 persons) compared to Whites. These racial/ethnic differences in the incidence and prevalence rates of diabetes are mirrored by disparities in diabetes-related complications.[4] Blacks have 2.5-fold and Hispanics have 3-fold increased risk of retinopathy compared to Whites.[5, 6] Although there are conflicting studies on the association between race/ethnicity and risk of cardiovascular disease (CVD) among patients with diabetes,[7–10] Blacks and Hispanics have higher rates of risk factors of CVD; that is, hypertension, uncontrolled blood glucose and lipid profiles compared to White individuals.[11–13].

Although primary prevention of diabetes is ideal, prevention of diabetes complications once a diagnosis is made is also paramount to decrease morbidity and mortality. Prevention efforts should be focused on the time that immediately follows a diagnosis of diabetes since the likelihood of complications at that time is low.[14, 15] Secondary prevention of microvascular and macrovascular complications of diabetes requires intervention early in the disease course by providing guideline-directed diabetes care for recently diagnosed individuals. For example, the American Diabetes Association (ADA) recommends an annual dilated eye exam for patients with diabetes.[16] However, the rate of guideline-directed eye screening was previously estimated to be only 60 % among individuals with diabetes.[17, 18] The ADA also recommends annual comprehensive foot examinations, as well as maintaining blood pressure control and lipid profile measurement every 5 years.[16].

While it is known that Hispanics and Blacks are disproportionately affected by diabetes complications, it remains unknown whether racial/ethnic disparities exist in the provision of guideline-directed measures of care early in the course of diabetes. The primary aim of this study is to assess differences in guideline-directed measures of diabetes care among Hispanics, Blacks, and Whites within 5 years of a diagnosis of diabetes. A secondary aim is to test whether socioeconomic status (SES), defined by family income, modifies the effect of race/ethnicity on the rate of guideline-directed prevention. We hypothesize that Hispanic and Black participants recently diagnosed with diabetes will have lower rates of guideline-directed care compared to Whites recently diagnosed with diabetes. We also further hypothesize that there is effect modification on race/ethnicity by SES.

## Methods

### Study population

We conducted an analysis of Hispanic, Black, and White participants in the National Health Interview Survey (NHIS) in the years 2011 through 2017. The NHIS is a nationally representative, cross-sectional, household interview survey conducted annually since 1957 intended to characterize and monitor the health of the non-institutionalized, civilian U.S. population. The NHIS survey design follows a stratified, multistage area probability design that allows the national representation of households, described previously.[19] For this analysis, inclusion criteria were: age 18 years of age or older and a diagnosis of diabetes within the prior five years, extracted from the data through the survey question “*Years since first diagnosed w/ diabetes.”* If participants responded yes to the Hispanic ethnicity question, they were categorized as Hispanic, otherwise they were categorized as either Black or White based on their response to the race question. Patients of Indian American, Chinese, Filipino, Asian Indian, other race or multiple race (no primary race selected) were excluded because the focus on this study was identifying differences among non-Hispanic White (*n*=4,289), non-Hispanic Black (*n*=1,337) and Hispanic (*n*=1,196) individuals.

### Outcomes

The primary outcome of this study was whether an individual had visited an eye specialist within the past 12 months, as captured by the question, *“Seen/talked to eye doctor, past 12 months*,*”* which had a dichotomous response (“yes”/”no”). Secondary outcomes included whether, in the past year, an individual visited a foot doctor as captured by the question *“Seen/talked to foot doctor, past 12 months”*; had cholesterol testing by the question *“Cholesterol checked by doctor/nurse/health professional, past 12 months”*, and had blood pressure checked by the question, *“Blood pressure checked by doctor/nurse/health professional, past 12 months”*.

### Socioeconomic status

We also hypothesized that SES, as defined by family income, was a modifier on the effect of race/ethnicity on the rate of yearly eye exam screening in individuals with diabetes. SES was specifically defined by income and dichotomized as either lower SES or higher SES depending on whether household income was below, or at or above 200 % of the federal poverty line.[20] We also conducted a sensitivity analysis with income above or below 400 % of the federal poverty line.

### Analysis

We first described demographic characteristics (age, sex, marital status and U.S. Census region), clinical characteristics (self-reported general health body mass index (BMI), number of comorbidities) and diabetes process measure outcomes (visited eye doctor, foot doctor, and had blood pressure or cholesterol checked within past 12 months) by race/ethnicity. We tested differences in the distribution of these characteristics by race/ethnicity via χ2 tests for categorical variables or via F-test for continuous variables.

The primary independent variables of interest were race/ethnicity, insurance coverage, SES, and the interaction of race/ ethnicity and SES. To estimate the associations between race/ethnicity and guideline-directed process measures for diabetes care, and whether SES or insurance status help explain these associations, we fit three sets of multivariate logistic regression models. Model 1 includes-baseline covariates in addition to race/ ethnicity, age, sex, self-reported general health status (excellent/very good vs. good/fair vs. poor), marital status (currently married vs. not currently married (widowed, divorced, separated, never married, living with partner, unknown marital status) and number of comorbidities associated with diabetes (count of ever told conditions: heart disease, angina, cancer, high cholesterol, kidney disease) [21]; Model 2 - includes Model 1 covariates and SES; and Model 3 - adds insurance coverage (categorized as having any insurance coverage or no coverage). The outcomes include the following guideline-directed process measures: visit to an eye doctor; visit to a foot doctor; blood pressure check; and cholesterol check. Model 1 covariates such as BMI, comorbidity count, and demographic variables were chosen *a priori* as potential confounders, and their distribution might be unequal across racial/ethnic groups. We conducted a sensitivity analysis by further adjusting the models with blood pressure check as an outcome for hypertension status (yes/no). On a secondary analysis, we added an interaction term between race/ethnicity and SES in Model 3 to test for effect modification of SES on the association between race/ethnicity and each outcome. The regressions were weighted by sampling weights to account for the complex sampling design of the NHIS. The p-values were calculated based on two-sided Wald tests, and *p*<0.05 was considered statistically significant. All analyses were performed using Stata 16 (StataCorp, College Station, TX). This study was approved by the Mass General Brigham Institutional Review Board.

## Results

The study population included *N*=7,341 individuals who were surveyed across 2011-2017. Whites were older than Blacks and Hispanics (mean 58.4 years vs. 54.8 vs. 52.0, *p*<0.001), more likely to be male, insured (93.2 % vs. 88.1 % vs. 75.9 %, *p*<0.001), and to report excellent/very good health (Table 1). Conversely, Blacks were more likely than Whites and Hispanics to report poor general health status (11.3 % vs. 9.1 % vs. 7.9 %, *p*<0.001).

**Table 1:** Description of individuals with a recent diagnosis of diabetes (within five years), by race/ethnicity

	Non-Hispanic Whites (weighted%)	Non-Hispanic Blacks (weighted%)	Hispanics (%)	p-value
N*	4289	1337	1196	
**Mean Age** (years)	58.4	54.8	52.0	<0.001
**Sex**				<0.001
Female	2124 (46.8)	801 (55.7)	660 (50.3)	
**Has health Insurance†**	3893 (93.2)	1124 (88.1)	854 (75.9)	<0.001
**General health status‡**				<0.001
Excellent/very good	1237 (28.7)	256 (19.4)	273 (25.9)	
Good/fair	2645 (62.1)	922 (69.3)	819 (66.2)	
Poor	404 (9.1)	158 (11.3)	104 (7.9)	
**Region**				<0.001
Northeast	718 (17.2)	172 (15.8)	165 (14.0)	
Midwest	1212 (29.0)	209 (17.0)	100 (9.8)	
South	1539 (38.2)	833 (58.3)	438 (37.6)	
West	820 (15.6)	123 (8.9)	493 (38.6)	
**Hypertension§**	3033 (70.1)	1076 (78.1)	716 (58.9)	<0.001
**Taking oral diabetes medication**	1093 (24.5)	339 (24.8)	327 (28.3)	0.07
**Taking insulin**	567 (12.2)	252 (17.2)	141 (11.5)	<0.001
**BMI||**				0.11
< 18.5	20 (0.4)	3 (0.2)	4 (0.4)	
18.5 – 24.9	423 (9.3)	129 (9.3)	127 (9.5)	
25.0 – 29.9	1166 (28.9)	346 (25.6)	367 (31.8)	
≥ 30.0	2468 (61.4)	808 (64.9)	642 (58.3)	
**Number of comorbidities¶**				<0.001
0	1216 (30.0)	517 (41.5)	551 (44.5)	
1	1493 (35.6)	433 (31.1)	406 (35.5)	
2-3	1273 (27.6)	318 (23.0)	205 (17.4)	
≥ 4	307 (6.8)	69 (4.4)	34 (2.5)	
**Married**	2111 (61.1)	392 (41.0)	548 (57.1)	<0.001
**Seen/talked to eye doctor (past 12 months)#**	2441 (57.1)	662 (48.8)	496 (41.3)	<0.001
Seen/talked to foot doctor (past 12 months)**	687 (16.5)	281 (18.9)	183 (13.5)	0.02
**BP check by health profession (past 12 months)††**	4116 (97.6)	1271 (96.3)	1060 (90.4)	<0.001
**Cholesterol check by health profession (past 12 months)‡‡**	3872 (92.9)	1203 (91.9)	997 (85.6)	<0.001

Notes:

*N=6,822 because 519 out of 7,341 participants have race/ethnicity data missing. Countsare not weighted, but percentages by race/ethnicity are weighted.

^†^ 271 participants missing data on insurance coverage

^‡^ 4 participants missing data on health status

^§^ 3 participants missing hypertension status information

^||^ 319 participants missing data on body mass index (BMI)

^¶^ Number of comorbidities related to diabetes and represents count of ever told about heartdisease, angina, cancer, high cholesterol, or kidney disease

^#^ 82 participants missing data on having a visit to an eye doctor in the past 12 months

^**^ 73 participants missing data on having a visit to a foot doctor in the past 12 months

^††^ 124 participants missing data on having blood pressure check by a health professional inthe past 12 months

^‡‡^ 183 participants missing data on having cholesterol check by a health professional in thepast 12 months

After adjustment for age, sex, oral diabetes medicine use, current insulin use, general health status, U.S. region, marital status, BMI, and number of comorbidities, Hispanics were less likely than White individuals to visit the eye doctor in the past year (Odds Ratio (OR) 0.65, 95 % Confidence Interval (CI) 0.54-0.79, *p*<0.001, Table 2). Though differences were attenuated, Hispanics remained less likely (OR 0.80, 95 % CI 0.65-0.99, *p* = 0.04) to report visiting the eye doctor in the past year compared to Whites even after further adjusting for SES and health insurance in Model 3. Although Black individuals had lower odds of visiting an eye doctor compared to White individuals in Model 1 (OR 0.81, 95 % CI 0.68-0.96, *p*=0.01), there was no significant difference in the odds of visiting an eye doctor between Blacks and Whites after adjustment for SES (Model 2) and health insurance (Model 3) (*p*=0.08 and *p*=0.12, respectively). Females were more likely than males (OR 1.37 (95 % CI, 1.18-1.58)) to report seeing an eye doctor in the prior year (*p*=<0.001 in Model 3). Compared to those without insurance coverage, those with any source of coverage were more likely (OR 2.46 (95 % CI, 1.90-3.18, *p*<0.001) to have visited the eye doctor in the past year.

**Table 2:** Association of patient characteristics with care received by individuals recently diagnosed with diabetes

In the past 12 months:	Eye doctor visit			Foot doctor visit			Blood Pressure check by health professional			Cholesterol check by health professional		
	**OR (95 % CI)**			**OR (95 % CI)**			**OR (95 % CI)**			**OR (95 % CI)**		
	Model 1*	Model 2*	Model 3*	Model 1*	Model 2*	Model 3*	Model 1*	Model 2*	Model 3*	Model 1*	Model 2*	Model 3*
**Age** (mean)	1.02 (1.02-1.03)‡	1.02 (1.02-1.03)‡	1.02 (1.01-1.03)‡	1.02 (1.02-1.03)‡	1.02 (1.02-1.03)‡	1.02 (1.01-1.03)‡	1.04 (1.02-1.05)‡	1.04 (1.02-1.05)‡	1.03 (1.01-1.05)‡	1.04 (1.03-1.05)‡	1.04 (1.03-1.05)‡	1.03 (1.02-1.04)‡
**Sex**												
Male	(ref)	(ref)	(ref)	(ref)	(ref)	(ref)	(ref)	(ref)	(ref)	(ref)	(ref)	(ref)
Female	1.29 (1.13-1.47)§	1.34 (1.16-1.55)‡	1.37 (1.18-1.58)‡	1.08 (0.91-1.28)	1.06 (0.88-1.27)	1.07 (0.88-1.29)	1.70 (1.20-2.42)§	1.79 (1.24-2.58)§	1.83 (1.26-2.66)§	1.14 (0.89-1.45)	1.14 (0.89-1.46)	1.13 (0.88-1.47)
**Race**												
Non-Hispanic white	(ref)	(ref)	(ref)	(ref)	(ref)	(ref)	(ref)	(ref)	(ref)	(ref)	(ref)	(ref)
Non-Hispanic black	0.81 (0.68-0.96)||	0.85 (0.71-1.02)	0.86 (0.72-1.04)	1.28 (1.03-1.61)||	1.38 (1.10-1.73)§	1.37 (1.08-1.74)§	0.75 (0.45-1.26)	0.86 (0.48-1.55)	0.82 (0.44-1.49)	1.14 (0.81-1.60)	1.33 (0.92-1.93)	1.30 (0.88-1.93)
Hispanic	0.65 (0.54-0.79)‡	0.74 (0.60-0.90)§	0.80 (0.65-0.99)||	0.99 (0.76-1.28)	1.06 (0.81-1.38)	1.11 (0.84-1.47)	0.39 (0.26-0.56)‡	0.38 (0.25-0.57)‡	0.42 (0.28-0.65)‡	0.66 (0.49-0.88)§	0.69 (0.51-0.93)||	0.81 (0.58-1.12)
**SES**												
High	-	(ref)	(ref)	-	(ref)	(ref)	-	(ref)	(ref)	-	(ref)	(ref)
Low	-	0.58 (0.50-0.68)‡	0.61 (0.52-0.71)‡	-	0.92 (0.76-1.12)	0.95 (0.77-1.17)	-	0.51 (0.34-0.76)§	0.60 (0.40-0.92)||	-	0.70 (0.54-0.90)||	0.81 (0.62-1.06)
**Has insurance**												
No	-	-	(ref)	-	-	(ref)	-	-	(ref)	-	-	(ref)
Yes	-	-	2.46 (1.90-3.18)‡	-	-	2.58 (1.69-3.95)‡	-	-	3.41 (2.31-5.04)‡	-	-	4.04 (2.99-5.44)‡

Notes:

*Model 1 was adjusted for sex, age, race/ethnicity, taking oral diabetes medicine, taking insulin, general health status, US. Census region, marital status, body mass index and number of comorbidities; Model 2 was adjusted for same covariates as Model 1 with the addition of adjustment for SES; Model 3 was adjusted for same covariates as Model 2 with the addition of adjustment for insurance status

‡*p*<0.001

§*p*<0.01

||*p*<0.05

In terms of secondary outcomes, in Model 1, Blacks were more likely (OR 1.28, 95 % CI 1.03-1.61, *p*=0.03) to have visited the foot doctor in the prior 12 months compared to Whites. The odds were more significant after adjustment for SES and insurance (OR 1.37, 95 % CI 1.08-1.74, *p* = 0.01). Compared to those without insurance, those with any insurance coverage were more likely (OR 2.58 (95 % CI 1.69-3.95, *p*<0.001)) to report visiting the foot specialist in the prior year. Hispanics recently diagnosed with diabetes were also less likely than Whites to report having their blood pressure checked by a health professional in the past 12 months (OR 0.42; (95 % CI 0.28-0.65, *p*<0.001)). Hispanic individuals were also significantly less likely to report a cholesterol check-up in the past 12 months compared to White individuals prior to adjustment for insurance coverage (*p*=0.004 for Model 1, *p*= 0.02 for Model 2). Compared to those without any insurance coverage, those with insurance were more likely to have a health professional check their blood pressure (OR 3.41 (95 % CI 2.31-5.04, *p*<0.001)), and cholesterol (OR 4.04 (95 % CI 2.99-5.44, *p*<0.001)). There were no significant interactions between race/ethnicity and SES. The sensitivity analyses using a higher income threshold for SES and further adjusting the models of blood pressure check as the outcome for hypertension showed similar trends as the main analyses (results not shown).

## Discussion

Our study showed that among participants recently diagnosed with diabetes, Hispanics had higher odds of being uninsured and lower odds of reporting receipt of guideline-directed care, such as yearly eye exams and blood pressure checks, compared to Whites. Black individuals were more likely than Whites to visit a foot doctor in the prior year. Our study is consistent with other studies that showed that uninsured, racial/ethnic minorities with diabetes were less likely to receive guideline-directed preventive care.[22, 23] Yet, to our knowledge, our study is one of the first to identify disparities in those recently diagnosed with diabetes.

There may be multiple factors that explain the racial/ethnic disparities in annual eye doctor visits observed in recently diagnosed individuals with diabetes. As seen in our study, lower SES and lack of health insurance may each have partial contributions for the observed disparities. Systemic and structural factors that lead to lower SES may prevent racial/ethnic minorities from purchasing health insurance to access preventive diabetes services; and high out-of-pocket-costs may deter them from seeking these services, even with insurance coverage. These disparities could have serious consequences as lack of insurance for Hispanics with diabetes is associated with higher rates of microvascular complications.[24] If lack of insurance mediates the association between Hispanic ethnicity and lower rates of annual eye doctor visits, Medicaid expansion and health insurance “subsidies” implemented through the Affordable Care Act could decrease disparities in preventive diabetes care. Continued policy efforts at the national and state levels are needed to ensure insurance coverage for racial/ethnic minorities [25] and care management services to help economically disadvantaged patients receive early preventive diabetes care.[26].

Hispanics and other racial/ethnic minorities are more likely to experience bias and stereotyping on the part of health care providers.[27] Perceived discrimination from primary care physicians (PCPs) is associated with decreased rates of routine visits at which preventive services are ordered.[28] Bias and discrimination may thus, also contribute to the observed differences in eye visit rates and blood pressure checks for Hispanics. Additional discrimination from providers could be due to language or insurance coverage barriers.[29] PCPs’ ineffective communication of treatment plans or not knowing where to refer in the absence of insurance coverage may delay diabetes preventive services and specialist referrals. These delays are particularly concerning if they occur recently after the diagnosis of diabetes, when prompt intervention is most likely to prevent morbidity.

Black individuals have lower rates of glycemic control than White individuals placing them at higher risk for microvascular complications, including foot ulcers and lower extremity amputations.[30–32] Given the observational nature of our study, it is difficult to determine the reason for the higher odds of foot doctor visits for Black individuals with diabetes. A study by Littman et al. showed a higher rate of foot self-inspection among groups at higher rates of lower extremity amputations such as Black individuals.[33] Authors also hypothesize that the association of higher odds of foot doctors visits among Black individuals may be due to race as a marker of disease severity and/or lower SES that could explain the paradoxical finding.[33].

We believe our study has two main strengths. First, our study used nationally representative data that oversamples minorities, which promotes the generalizability of our findings to the U.S. population and allows for a large enough sample size to look at racial/ethnic disparities. Second, these data allow us to specifically examine racial/ethnic disparities in diabetes care early in the course of diabetes when complications are preventable.

However, this study is not without limitations. First, the responses to the survey were self-reported, which could introduce response bias from study participants. Response bias may have led to inaccurate participant reporting the receipt of diabetes guideline-directed services. Yet, our results do not indicate that potential reporting inaccuracies would favor any racial/ethnic group. Second, participants recently diagnosed with diabetes might have differed in the course of their disease process and already had serious diabetes complications that differed by race/ethnicity.[34] While differences in diabetes course and severity by race/ethnicity at the time of diagnosis are possible, all participants are within five years of their diabetes diagnosis which possibly attenuates the impact of those differences. Third, the disparities observed in this study may be a reflection of broader disparities in patients with diabetes, and not specific to individuals recently diagnosed with diabetes. Even if the results of our study reflect broader disparities in diabetes, demonstrating disparities in the period soon after the diagnosis of diabetes may be a catalyst for action in a highly-opportune time to mitigate them. Fourth, data on timing of outcomes was only available within a year prior to the survey and not immediately following diagnosis for those diagnosed several years prior to taking the survey. That is a limitation of the data and cross-sectional study design that does not diminish the significance of our findings. Our study objective was to measure yearly receipt of guideline-directed care instead of care received immediately after diagnosis. Fifth, the variable for having seen/talked to an eye doctor in the past year may capture consultations for reasons other than a diabetic retinal eye exam or a visit for diabetes-related eye complications. While that variable may underrepresent diabetic retinal eye exams, in clinical practice nearly all individuals with diabetes who undergo an eye exam will be screened or undergo surveillance for diabetes-related complications. Sixth, there was limited variability in participants receiving blood pressure and cholesterol checks, which reduces the power to detect meaningful differences across racial/ethnic groups. Lastly, there may be racial/ethnic differences in the timing of diagnosis relative to the period in which outcomes were observed that were not accounted for in the analysis. Future work should evaluate PCPs attitudes and biases and patient navigation resources when treating racial/ethnic minorities with diabetes. Future work may also include additional control groups to better disentangle the source of the disparities observed in our study.

## Conclusions

In conclusion, Hispanics recently diagnosed with diabetes do not receive guideline directed eye care and blood pressure measurement compared to Whites. Insurance status and poverty may contribute to these differences. Future work should consider physician-level factors such as cultural and language sensitivity, and presence of implicit and explicit bias and their relation to providing guideline-directed care.

## Data Availability

The datasets generated and/or analyzed during the current study are available from the U.S. Centers for Disease Control and Prevention website, https://www.cdc.gov/nchs/nhis/data-questionnaires-documentation.htm.

## Abbreviations

U.S.: United States of America
CDC: Centers for Disease Control and Prevention
CVD: cardiovascular disease
ADA: American Diabetes Association
SES: socioeconomic status
NHIS: National Health Interview Survey
BMI: body mass index
OR: odds ratio
CI: confidence interval
PCPs: primary care physicians
